# Anorexia nervosa with subsequent onset of schizophrenia: A case report and literature review

**DOI:** 10.1002/pcn5.70172

**Published:** 2025-07-30

**Authors:** Yuhei Suzuki, Akiko Sato, Yuhei Mori, Risa Shishido, Shuntaro Itagaki, Itaru Miura

**Affiliations:** ^1^ Department of Neuropsychiatry, School of Medicine Fukushima Medical University Fukushima Japan

**Keywords:** anorexia nervosa, eating disorders, prodromal psychosis, schizophrenia, self‐efficacy

## Abstract

**Background:**

Anorexia nervosa (AN) is closely associated with schizophrenia. A recent meta‐analysis reported that the comorbidity of eating and psychotic disorders is approximately 8%, suggesting a potential link between the two. However, the characteristics and management of AN patients who later develop schizophrenia remain insufficiently explored.

**Case Presentation:**

We report a 17‐year‐old woman with AN since age 11. Despite multiple hospitalizations and behavioral interventions, she persisted in restrictive eating. Additionally, she exhibited emotional dysregulation, irritability, and self‐injurious behavior, which led to low‐dose risperidone initiation. At age 16, she developed persecutory delusions and auditory hallucinations, resulting in a diagnosis of schizophrenia. Notably, her disordered eating resolved following the onset of psychotic symptoms. With a higher dose of risperidone, her psychosis improved, and she remained stable without relapse or significant weight loss for over a year.

**Conclusion:**

This case illustrates the potential relationship between AN and schizophrenia, suggesting that AN may represent a prodromal phase of schizophrenia. Our literature review indicates that the timing and nature of psychotic symptoms can aid in differentiating psychosis linked to AN from schizophrenia. The case also suggests that AN and schizophrenia may exert mutually inhibitory effects on each other, potentially influencing their respective courses.

## BACKGROUND

Anorexia nervosa (AN) and schizophrenia are closely associated, as supported by genetic and epidemiological evidence.^1^, ^2^ Shared psychosocial risk factors—such as childhood adversity, abuse, and trauma—also contribute to both disorders. A recent meta‐analysis reported an 8% comorbidity rate between eating disorders and psychotic disorders.^3^ Furthermore, about 10% of schizophrenia patients have a history of eating disorders, and approximately half of them have AN. This suggests that AN may, in some cases, represent a prodromal phase of schizophrenia.^4^

Reports describe AN cases accompanied by psychotic symptoms or followed by schizophrenia, with some suggesting that schizophrenia onset alters eating behaviors.^5^ One hypothesis is that disordered eating behaviors may function as a defense against a fragile and disintegrated self‐state.^6^ However, the mechanisms driving these changes remain unclear. Moreover, distinguishing psychosis as part of AN from emerging schizophrenia poses diagnostic challenges.

Here, we present a case of AN followed by schizophrenia, with remission of disordered eating behavior upon psychosis onset, and review the literature on similar cases.

## CASE PRESENTATION

The patient was a 17‐year‐old woman, the second of three siblings, and a twin. Her co‐twin and other family members had no history of psychiatric disorders. She had no history of perinatal complications, developmental delays, or use of psychoactive substances or illicit drugs.

**Figure 1.**
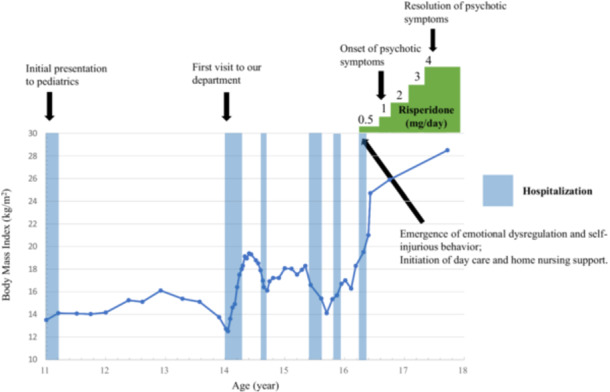
Treatment timeline, body mass index, and psychotic symptom progression.

The timeline of the clinical course, including changes in BMI, psychotic symptoms, and treatment interventions, is shown in Figure 1.

At age 11, she began restricting food and engaging in excessive physical activity, including running for more than 3 h per day, after peers commented on her body shape, and lost nearly 9 kg in a month. She was hospitalized in the pediatric department for medical treatment.

Her symptoms persisted after discharge, leading to her first visit to our department at age 14 with a BMI of 12.7 kg/m². She denied being underweight and refused weight restoration treatment. Based on her presentation, which was characterized by severe dietary restriction, hyperactivity, distorted body image, and the absence of bingeing or purging behavior, she was diagnosed with restricting‐type AN.

Despite regaining weight after nutritional and behavioral therapy, she experienced relapses, requiring repeated hospitalizations. Over time, she became less cooperative, necessitating nasogastric feeding and behavioral restrictions, including physical restraint to prevent self‐removal of the nasogastric tube and excessive physical activity. She controlled family meals and pressured her mother to reduce portions, while her overwhelmed parents alternated between ignoring her behavior and complying with her demands.

She exhibited emotional dysregulation, including impulsive self‐injurious behaviors and marked irritability. During her fifth hospitalization, risperidone (1 mg) was initiated to help manage these symptoms. After middle school, she joined a daycare program and received home nursing care.

At 16, she developed persecutory delusions (“someone is watching me”) and auditory hallucinations commanding her death. She withdrew socially, and despite adequate nutrition (BMI approximately 20), she developed psychotic symptoms during this period.

She met DSM‐5‐TR criteria for schizophrenia, with persistent hallucinations, delusions, and negative symptoms accompanied by marked social impairment for over 6 months. She had no history of substance or illicit drug use, and no mood episodes, such as depression or mania, were observed to coincide with the psychotic symptoms. Therefore, schizoaffective disorder and substance/medication‐induced psychotic disorder were ruled out.

In addition, her strong preoccupation with food, irritability, and the autistic‐like behavior observed after the onset of psychosis raised the possibility of comorbid autism spectrum disorder or a communication disorder. However, given her good social functioning and relatively adequate academic adjustment before the onset of anorexia nervosa, and the fact that these symptoms appeared only after the onset of AN, these diagnoses were not supported.

Risperidone was gradually increased to 4 mg over several months, which led to improvement in psychosis. Remarkably, disordered eating behavior resolved, and BMI stabilized at approximately 25 kg/m². She has remained well without weight loss relapse for over 15 months.

## DISCUSSION

The patient developed schizophrenia 6 years after AN onset, with emotional dysregulation and self‐injurious behaviors serving as prodromal features. Notably, her disordered eating behaviors subsided concomitantly with the emergence of psychotic symptoms. Treatment with antipsychotics led to an improvement in psychotic symptoms, and disordered eating behaviors resolved around the same time that the psychotic symptoms emerged. We made a final diagnosis of schizophrenia, and antipsychotic medication was continued. However, as there has been no relapse since the initial episode, and psychotic symptoms have improved alongside eating behaviors, careful follow‐up is warranted to confirm this diagnosis.

Our systematic literature review identified six cases of AN patients subsequently diagnosed with schizophrenia. A search was conducted on PubMed using the keywords “(psychosis OR schizophrenia) AND (anorexia nervosa OR eating disorders)”, and focusing on case reports. The search included studies published until March 31, 2025. Cases with bulimia nervosa, avoidant/restrictive food intake disorder, organic comorbidities, psychosis preceding the onset of AN, or AN secondary to schizophrenia were excluded. Initially, we identified eight case reports of patients with schizophrenia during AN treatment. However, two cases were excluded because of comorbid organic brain diseases (Table 1).^6^, ^7^, ^8^, ^9^, ^10^, ^11^ In one case, disordered eating behavior resolved at the onset of psychosis.^8^ Another patient relapsed into disordered eating after the positive symptoms resolved.^10^ After antipsychotic treatment, two patients showed improvement in both eating behaviors and psychosis, while two others showed partial improvement with persistent psychiatric and eating disturbances.

**Table 1.** A review of case reports on schizophrenia onset during the course of anorexia nervosa.

Authors, publication year	Diagnosis	Sex	Onset age of AN (years)	Onset age of SZ	Content of psychosis	Treatment and clinical course
Shiraishi et al., 1992 (Case 1)	AN, SZ	F	12	14	Delusions of external control (believing that a woman was ordering her actions) and possession by various animals.	The patient was treated with clocapramine, leading to a gradual improvement in positive symptoms. However, at the time of reporting, psychiatric symptoms remained insufficiently stabilized.
Yamashita et al., 1994	AN, SZ	F	13	14	Persecutory delusions, auditory hallucinations, and bizarre delusions (belief that people can survive without food).	Following the onset of psychotic symptoms, disordered eating behaviors improved. The patient was treated with haloperidol, which led to the resolution of the psychotic symptoms. No relapse of disordered eating behaviors was observed thereafter.
Hugo et al., 1997 (Case 4)	AN (bulimic type), SZ	F	18	19	Believed that she had a blocked gastrointestinal tract and reported olfactory hallucinations.	Initially diagnosed with primary depression, the patient underwent ECT and antipsychotic treatment. Despite near‐normal weight recovery, the psychotic symptoms persisted. Eating disorder treatment was suspended, and upon discharge (59 kg), bulimic features predominated.
Kirary et al., 2003	AN (bulimic type), SZ	M	23	23	Suicidal ideation and bizarre delusions, but no auditory or visual hallucinations.	Olanzapine treatment led to improvements in both psychotic symptoms and disordered eating behaviors. However, after discontinuing the treatment, both symptoms relapsed.
Cinemre et al., 2007	AN, SZ	M	14	18	Paranoid delusions (fear of hidden cameras, belief that TV conversations were directed at him), thought broadcasting, and disorganized thinking.	The patient was treated with olanzapine and quetiapine. When positive symptoms improved, disordered eating behaviors appeared to relapse.
Crisan et al., 2021	AN, SZ	F	15	22	Auditory hallucinations (voices giving orders), paranoid delusions, and destructive behavior (breaking glass).	Olanzapine treatment resulted in improvements in both psychotic symptoms and disordered eating behaviors.
Suzuki et al., 2025 (present case)	AN, SZ	F	11	17	Auditory hallucinations (insults and voices conversing) and persecutory delusions of being watched.	From the onset of psychotic symptoms, disordered eating behaviors improved. Treatment with risperidone led to the resolution of hallucinations and delusions.

Abbreviations: AN, anorexia nervosa; SZ, schizophrenia.

In most cases, schizophrenia was diagnosed several years after the onset of AN. In contrast, in cases where psychotic symptoms were present but did not meet the criteria for schizophrenia, AN and the psychotic symptoms tended to begin around the same time.^12^, ^13^ An average interval of 7 years in males and 4 years in females between the onset of an eating disorder and the diagnosis of schizophrenia suggests that AN may, in some cases, represent a prodromal phase of schizophrenia.^4^ However, distinguishing schizophrenia from AN‐related psychotic symptoms remains challenging. Crișan et al. reported cases where AN and schizophrenia coexisted, highlighting the difficulty in differentiating delusions from overvalued ideas linked to food restriction.^11^ Prior studies suggest that the nature of delusions and hallucinations may help differentiate the two conditions: patients with schizophrenia often exhibit delusions unrelated to food, while those with AN‐related psychosis typically have delusions tied to eating, such as believing they can survive without food or hearing divine commands to avoid eating.^13^, ^14^ However, given the limited evidence, this distinction is not yet a reliable diagnostic criterion.

All six patients in this review received antipsychotics, with five showing improvement. Although meta‐analyses have found no clear effects of antipsychotics on body weight or related outcomes in females with AN, they may still benefit AN patients with psychotic features or comorbid schizophrenia.^14^, ^15^, ^16^

In contrast, one study described a case where antipsychotics were initially ineffective for AN‐related psychotic symptoms, but psychosis improved after weight restoration.^13^ This suggests that, unlike schizophrenia and primary psychotic disorders, psychosis in AN may, in certain cases, respond better to nutritional rehabilitation than to antipsychotic treatment. This may reflect the role of chronic malnutrition in increasing the risk of schizophrenia by impairing brain development through mechanisms such as oxidative stress and neuroinflammation.^17^ Therefore, nutritional status at psychosis onset may influence treatment priorities, with nutritional rehabilitation taking precedence in some cases. In the present case, prolonged malnutrition during early childhood may have adversely affected brain development and contributed to the later onset of schizophrenia.

Our findings suggest that in some cases, schizophrenia onset in AN patients is associated with changes in disordered eating behaviors. It has been proposed that prodromal schizophrenia, marked by a loss of self‐agency and control, may drive food restriction as a coping mechanism, with disordered eating functioning as a defense against a fragile or disintegrated self‐state.^5^, ^6^

This case also highlights that AN and schizophrenia may share psychosocial risk factors. The patient's AN was triggered by a classmate's comment about body shape, but subsequent social isolation and excessive efforts to adapt socially may have contributed to both AN symptoms and later schizophrenia. Previous studies have identified childhood adversity, such as abuse and bullying, as shared risk factors for both conditions.^18^, ^19^ Following the onset of emotional dysregulation, including impulsivity, self‐injurious behaviors, and irritability, the patient began attending day care and receiving home nursing support, which provided a more individualized and supportive environment. These supports may have also contributed to the AN symptom remission.

In this case, low‐dose risperidone was initiated before the onset of schizophrenia. However, psychotic symptoms persisted, leading to a diagnosis of schizophrenia. Her presentation prior to the onset of psychotic symptoms—characterized by anorexia nervosa, emotional dysregulation, and self‐injurious behaviors—may reflect features consistent with an at‐risk mental state (ARMS), or at least a condition overlapping with it. Although disordered eating behaviors themselves are not explicitly included in the current ARMS diagnostic criteria, several studies have reported a high rate of co‐occurrence between eating disorders and ARMS.^20^ Moreover, affective dysregulation is frequently observed among individuals meeting ARMS criteria and has been proposed as a mediator in the progression from stress or trauma to the onset of psychosis.^21^ These findings suggest that such comorbid features may serve as clinical indicators of ARMS, even in the absence of typical prodromal psychotic symptoms. Studies suggest that antipsychotics have limited effect in preventing the transition to psychosis; therefore, psychosocial interventions, including cognitive behavioral therapy‐based approaches, are recommended.^22^ In addition to pharmacological treatment, comprehensive management of eating disorders and psychosocial support may be critical in preventing psychosis.

This study has several limitations. As a review of case reports, its generalizability is limited and subject to selection bias. Furthermore, some patients with psychotic symptoms during AN may meet diagnostic criteria for schizophrenia, but are not formally diagnosed or reported. Nevertheless, to our knowledge, this is the first study to provide a detailed longitudinal assessment of cases where schizophrenia developed from AN, examining both treatment response and the trajectory of disordered eating behavior.

## CONCLUSION

This case highlights the complex relationship between AN and schizophrenia, suggesting that AN may serve as a prodromal phase of schizophrenia and that the onset of psychosis in AN may alter eating behaviors.

Our review indicates that the interval between AN onset and psychosis, as well as the nature of psychotic symptoms, may help differentiate psychosis associated with AN from primary psychotic disorders such as schizophrenia.

Additionally, our findings suggest that psychotic symptoms and AN‐related eating disorders may have a mutually inhibitory relationship, influencing the clinical course of both conditions. Shared environmental factors may also contribute to the development of both disorders, underscoring the importance of evaluating and addressing these factors in management.

Further research is needed to clarify the mechanisms underlying the co‐occurrence or sequential development of AN and schizophrenia, to improve diagnostic accuracy, and to tailor treatment strategies for patients with AN who develop psychotic symptoms.

## AUTHOR CONTRIBUTIONS

Yuhei Suzuki treated the patient, reviewed the literature, and drafted the manuscript. Itaru Miura supervised the study. All authors reviewed the draft and approved the manuscript.

## CONFLICT OF INTEREST STATEMENT

The authors declare no conflicts of interest.

## ETHICS APPROVAL STATEMENT

The patient and her family provided consent for publication of this case report. The report complies with the rules of the Ethics Committee of Fukushima Medical University Hospital and the provisions of the Declaration of Helsinki.

## PATIENT CONSENT STATEMENT

The patient provided written informed consent for publication of this report.

## CLINICAL TRIAL REGISTRATION

N/A.

## Data Availability

Data sharing is not applicable to this article as no datasets were generated or analyzed during the current study.
